# Dual Energy CT-Derived Liver Extracellular Volume Fraction for Assessing Liver Functional Reserve in Patients with Liver Cirrhosis

**DOI:** 10.3390/medicina61091561

**Published:** 2025-08-30

**Authors:** Seok Jin Hong, Ji Eun Kim, Jae Min Cho, Ho Cheol Choi, Mi Jung Park, Hye Young Choi, Hwa Seon Shin, Jung Ho Won, Wonjeong Yang, Hyun Ok Kim

**Affiliations:** 1 Department of Radiology, Institute of Medical Science, Gyeongsang National University College of Medicine and Gyeongsang National University Hospital, Jinju-si 52727, Republic of Korea; iamgoodbaba@hanmail.net (S.J.H.); jmcho@gnu.ac.kr (J.M.C.); jaro2@hanmail.net (H.C.C.); pichola@naver.com (M.J.P.); whitecoc@hanmail.net (H.Y.C.); hshin0315@gmail.com (H.S.S.); circlehoya@naver.com (J.H.W.); piktors@naver.com (W.Y.); 2 Department of Internal Medicine, Institute of Medical Science, Gyeongsang National University College of Medicine and Gyeongsang National University Hospital, Jinju-si 52727, Republic of Korea; galleonok@hanmail.net

**Keywords:** dual-energy CT, extracellular volume fraction, liver function, liver cirrhosis

## Abstract

*Background and Objectives*: The extracellular volume fraction (fECV) of the liver, as measured by contrast-enhanced computed tomography (CT), has been shown to correlate closely with the histological stages of hepatic fibrosis. This study aimed to investigate the diagnostic performance of a liver extracellular volume fraction derived from dual-energy CT (DECT) for evaluating liver functional reserve based on the Child–Pugh class in cirrhotic patients, compared with other noninvasive markers. *Materials and Methods*: This retrospective study included 258 patients with liver cirrhosis who underwent contrast-enhanced DECT. The fECV was measured using iodine maps derived from equilibrium phase images obtained 3 min after contrast injection at 100/140 Sn kVp. Statistical analyses included Welch’s ANOVA with post hoc tests, Spearman’s correlation, and ROC analysis. The area under the curve (AUC) was compared among fECV and other noninvasive markers (aspartate transaminase to platelet ratio index [APRI], Fibrosis-4 [FIB-4], and model for end-stage liver disease [MELD]) using DeLong’s test. Intra- and interobserver reliability of fECV was assessed with the intraclass correlation coefficient (ICC). The area under the receiver operating characteristic curve (AUC) for differentiating Child–Pugh classes was compared among the fECV and other noninvasive markers (aspartate transaminase to platelet ratio index [APRI], Fibrosis-4 [FIB-4], and model for end-stage liver disease [MELD]). *Results*: The fECV increased significantly with advancing Child–Pugh classes (*p* < 0.001), showing a moderate correlation with Child–Pugh class (r = 0.53). The mean differences in fECV among the Child–Pugh classes were 8.88 between A and B (95% confidence interval [CI], 5.85–11.92; *p* < 0.001) and 7.42 between B and C (95% CI, 1.92–12.91: *p* < 0.001). The AUC for differentiating Child–Pugh classes A and B demonstrated no significant differences among the fECV (0.84), APRI (0.83, *p* > 0.99) and FIB-4 (0.83, *p* > 0.99), except for MELD, which had a significantly higher AUC (0.94, *p* = 0.047). For differentiating Child-Pugh classes B and C, the fECV demonstrated a significantly higher AUC (0.78), compared with FIB-4 (0.50, *p* = 0.038) and APRI (0.49, *p* = 0.037), whereas no significant difference was observed between fECV and MELD (0.92, *p* = 0.12). The intra- and interobserver reliabilities of the fECV measurements were excellent (ICC, 0.93; 95% CI, 0.91–0.95 and 0.91; 95% CI, 0.88–0.92, respectively). *Conclusions*: DECT derived fECV is a useful noninvasive marker for assessing liver functional reserve based on the Child–Pugh classification.

## 1. Introduction

Liver cirrhosis is characterized histologically by widespread nodular regeneration surrounded by dense fibrous septa, along with the progressive loss of normal hepatic parenchyma [1]. Major etiologies such as chronic alcohol consumption, hepatitis B virus, hepatitis C virus, metabolic dysfunction–associated steatotic liver disease, and autoimmune hepatitis have been identified [1]. Cirrhosis disrupts normal liver architecture and alters portal blood flow, leading to portal hypertension and hepatic dysfunction. These changes can result in various complications that not only impair patients’ quality of life but also increase the risk of serious outcomes such as hepatocellular carcinoma (HCC) and variceal bleeding, ultimately contributing to a higher mortality rate compared to patients without cirrhosis [1]. In decompensated liver cirrhosis, where liver function is significantly impaired, major complications occur more frequently than in compensated cirrhosis, where liver function is relatively preserved [1]. Thus, accurate evaluation of hepatic functional reserve is essential in cirrhotic patients [1,2,3].

Liver stiffness has gained attention as a surrogate marker for predicting the progression of cirrhosis, development of HCC, and mortality [2]. Liver stiffness values obtained through FibroScan^®^ (Echosens, Paris, France), ultrasound (US) elastography, and magnetic resonance (MR) elastography have demonstrated a strong correlation with histologic fibrosis stages and liver function [4,5,6,7,8,9]. However, each elastography technique has its limitations. FibroScan is considered a “blind” method, which may include unintended structures such as vessels or focal hepatic lesions within the measurement area, potentially affecting the results [10]. US elastography is subject to operator dependency and inter-vendor variability in measurement standards [11]. Although MR elastography offers the highest accuracy and reproducibility, its clinical application is limited by high cost and relatively low accessibility [4,12]. Moreover, all elastography methods face significant limitations in patients with obesity or ascites [4,5,6,10,11,12].

Computed tomography (CT) is widely used for HCC surveillance and the evaluation of cirrhosis-related complications [13,14]. Fibrosis leads to expansion of the extravascular extracellular space in the liver due to collagen deposition, and contrast material used in CT imaging eventually equilibrates between the intravascular and extracellular compartments. Based on this principle, recent studies have demonstrated that extracellular volume fraction (fECV) measured by contrast-enhanced CT correlates closely with histological stages of hepatic fibrosis [15,16,17,18,19,20]. fECV assessment using CT has practical advantages, including applicability in patients with obesity or ascites and the ability to place regions of interest directly in desired liver parenchyma, thereby overcoming the field-of-view limitations associated with FibroScan [15,16,17]. Furthermore, CT is more accessible and less expensive than MR-based techniques. Notably, recent studies have shown that fECV values derived from dual-energy CT (DECT) acquired in the equilibrium phase can provide similar or superior diagnostic performance for liver fibrosis compared to single-energy CT performed at a delayed phase [21].

Because progression of fibrosis inevitably translates into deterioration of hepatic function, indices that assess fibrosis are closely associated with biomarkers reflecting he-patic function reserve [22,23]. The Child–Pugh score is a widely used clinical tool for evaluating hepatic functional reserve and predicting prognosis in cirrhosis [22,23]. Several serum biomarkers that reflect hepatic fibrosis have also been utilized to evaluate liver function and predict prognosis [24,25,26,27,28]. There have been several studies using fECV derived from dual-energy or spectral CT to evaluate the stage of hepatic fibrosis and the severity of liver cirrhosis [21,29,30]. However, to date, no study has evaluated its clinical utility in assessing liver function according to the Child–Pugh classification under the following clinically relevant conditions: (1) fECV correction using both hematocrit and aortic enhancement, (2) application of dual-source dual-energy CT, and (3) use of a clinically feasible equilibrium phase at 180 s.

Therefore, the aim of this study was to investigate the correlation between liver function based on the Child–Pugh classification and fECV derived from dual-source dual-energy CT. Additionally, we compared its performance with other noninvasive markers and evaluated its predictive value in differentiating among Child–Pugh classes.

## 2. Materials and Methods

### 2.1. Patient

**Figure 1:**
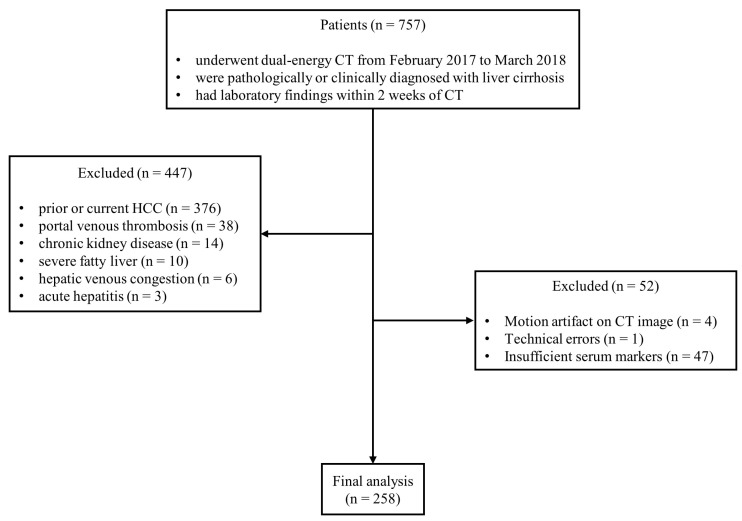
Flowchart of patient enrollment and exclusion criteria. (Abbreviations: CT, computed tomography; HCC, hepatocellular carcinoma).

From February 2017 to March 2018, we included patients who met the following eligibility criteria based on our radiological and electric medical record database: (1) dual-source DECT imaging with equilibrium phase scanning in dual-energy mode were performed (2) liver cirrhosis was pathologically or clinically proven (3) serum markers was obtained within 2 weeks of CT imaging. Patients were excluded if either of the following criteria were met: (1) present HCC identified on CT or a prior diagnosis of HCC (2) portal vein thrombosis (3) chronic kidney disease (4) severe fatty liver (5) hepatic venous congestion (6) acute hepatitis (7) motion artifacts on CT image (8) incomplete laboratory results (Figure 1). One additional case with a technical error was excluded. Liver cirrhosis was confirmed either by liver biopsy (F4 fibrosis, *n* = 9) or by a combination of clinical findings (an identified etiology of cirrhosis and serum markers suggestive of cirrhosis, with or without hepatic encephalopathy or varices on endoscopy within 6 months of the initial CT imaging) and CT imaging features (liver surface nodularity and signs of portal hypertension, such as splenomegaly, portosystemic collaterals, or ascites) (*n* = 239). Clinical and laboratory data were collected for all included patients through a review of electronic medical records. The collected clinical variables included age, sex, etiology of liver cirrhosis, presence of ascites, and hepatic encephalopathy. The laboratory parameters included serum sodium, albumin, platelet count, total bilirubin, aspartate aminotransferase (AST), alanine aminotransferase (ALT), serum creatinine, and international normalized ratio (INR). Based on this information, the Child–Pugh classification, fibrosis-4 (FIB-4) index, aspartate aminotransferase to platelet ratio index (APRI), and model for end-stage liver disease (MELD) score were calculated for each patient [24,25,26,27].

### 2.2. CT Acquisition

All patients underwent four-phase liver CT using a 128-slice dual-source CT scanner (Definition Flash, VA44A; Siemens Healthineers, Erlangen, Germany). The noncontrast and portal venous phases were acquired with single-energy mode using automated tube voltage selection (CARE kV, Siemens Healthineers, Erlangen, Germany). Scanning parameters included a reference voltage of 120 kVp, tube current of 120 and 180 mAs, section collimation of 128 × 0.6 mm with a z-flying focal spot, a rotation time of 0.5 s, and a helical pitch of 0.75. For the late arterial and equilibrium phases, dual-energy mode was employed with 100/140 Sn kVp. Parameters included reference tube currents of 130 and 100 mAs, section collimation of 64 × 2 × 0.6 mm, a rotation time of 0.33 s, and a helical pitch of 0.7. In both modes, automatic current modulation (CARE Dose 4D, Siemens) was applied. The scan covered the area from the lung bases to the iliac crest. The median volumetric CT dose index (CTDIvol) was 24.8 mGy, and the median dose-length product was 919.0 mGy·cm. For a single postcontrast phase, CTDIvol was comparable between single- and dual-energy protocols (6.78 vs. 6.79 mAs, respectively). Iodinated contrast agent (Iopamidol, 370 mg iodine/mL; Dasol Life Science, Seoul, Republic of Korea) was administered via an antecubital vein at 1.5 mL/kg body weight with a flow rate of 3 mL/s. Late arterial-phase imaging was triggered 17 s after the aortic enhancement reached 100 HU at the level of the hepatic dome, using an automatic bolus-tracking system (CARE Bolus CT, Siemens). Portal venous and equilibrium phases were acquired at 60 and 180 s post-injection, respectively. Reconstruction of the DECT data was performed with 1.5 mm slice thickness and 1.0 mm interval using the Q40f kernel and sinogram-affirmed iterative reconstruction (SAFIRE, strength 2; Siemens). Processed images were transferred to a dedicated analysis platform (syngo.via, version VB30A; Siemens Healthineers, Erlangen, Germany).

### 2.3. Image Analysis

**Figure 2:**
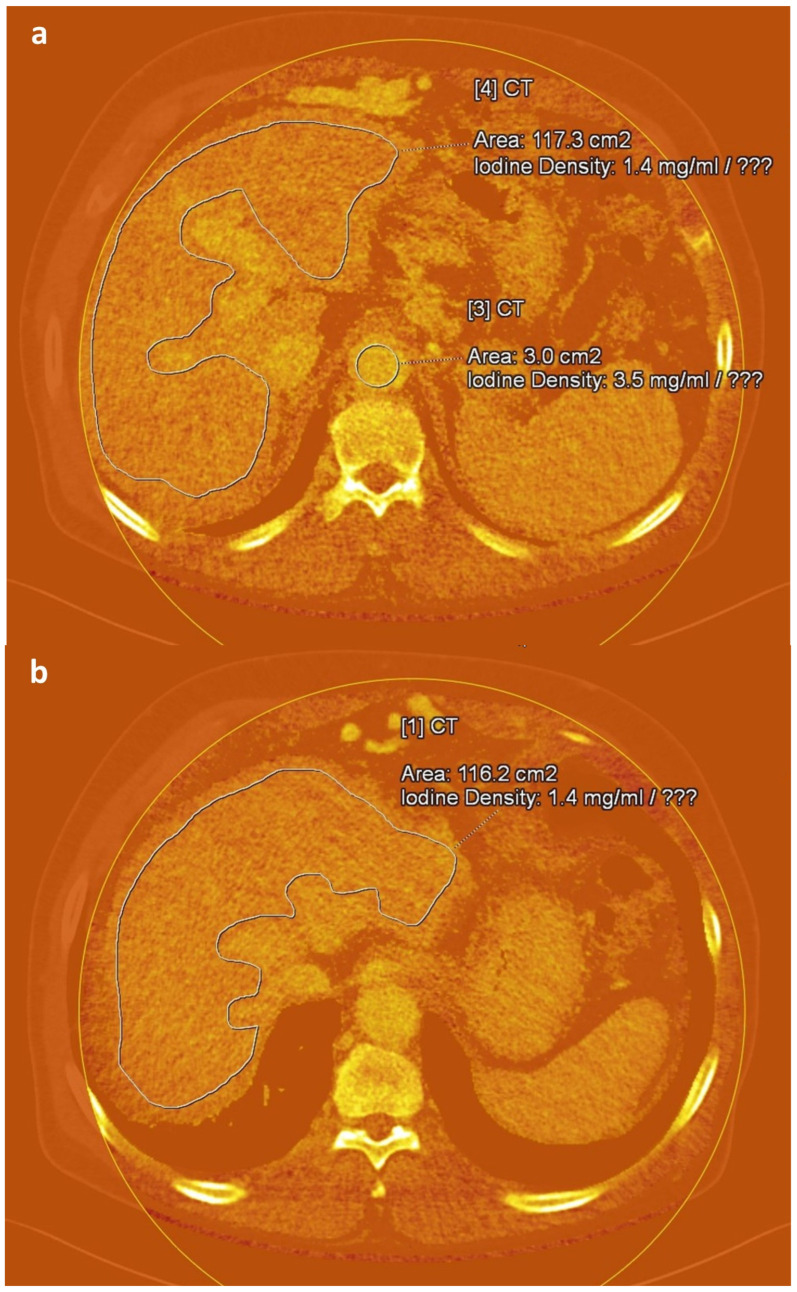
Axial iodine maps generated from equilibrium-phase images obtained 3 min after contrast administration at 100/140 Sn kVp in a 72-year-old man with alcohol-induced liver cirrhosis. Freehand (**a**,**b**) and circular (**a**) regions of interest are delineated in the liver and aorta, respectively, at different anatomical levels on the iodine maps.

An abdominal radiologist (J.E.K., 17 years of experience in CT interpretation), blinded to all clinical information, manually delineated regions of interest (ROIs) on iodine maps reconstructed from equilibrium-phase images using the Liver VNC tool provided in the syngo.via software platform (version VB60S; Siemens Healthineers, Erlangen, Germany). For each case, three free-hand ROIs were placed along the margin of the liver at the anatomical levels of the intrahepatic inferior vena cava confluence, portal vein hilum, and gallbladder fossa, while carefully avoiding major vessels and focal hepatic lesions. To reduce variability due to scan timing, three circular ROIs were additionally positioned in the aorta at the origins of the celiac trunk, superior mesenteric artery, and renal arteries [31] (Figure 2). The average iodine concentration values derived from these ROIs were used to represent hepatic iodine content (IC_Liver) and aortic iodine content (IC_Aorta). Based on these values, fECV score was computed using the following formula [18,19,31]:

fECV score = (IC_Liver/IC_Aorta) × (100 − hematocrit [%])

For evaluation of interobserver agreement, an abdominal radiologist (SJH, 8 years of experience in CT interpretation) independently calculated fECV scores in separate sessions using the same software. To assess intraobserver reliability, the original radiologist (JEK) repeated the measurements four weeks after the initial session.

### 2.4. Statistical Analysis

Continuous variables were expressed as mean ± standard deviation (SD) or median with interquartile range (IQR), depending on data distribution. Categorical variables were presented as frequencies and percentages. To compare clinical and laboratory variables across Child–Pugh classes A, B, and C, Welch’s ANOVA was used for continuous variables due to unequal variances and sample sizes, followed by Games–Howell post hoc tests to assess pairwise differences. Correlations between each noninvasive marker (fECV, FIB-4, APRI, and MELD) and Child–Pugh class were evaluated using Spearman’s rank correlation coefficient. To assess the diagnostic performance of each marker in differentiating between Child–Pugh classes (A vs. B, B vs. C), the area under the receiver operating characteristic curve (AUC) was calculated. Differences in AUCs between markers were compared using DeLong’s test, with Holm correction applied for multiple comparisons. Intra- and interobserver reliability of fECV score measurements was evaluated using the intraclass correlation coefficient (ICC), calculated with a two-way random-effects model for single measurements and absolute agreement. A *p*-value < 0.05 was considered statistically significant. All statistical analyses were performed using R (version 4.4.3; R Foundation for Statistical Computing, Vienna, Austria).

## 3. Results

### 3.1. Patient Characteristics

A total of 258 patients was included in our study. Among them, 207 patients were classified Child–Pugh class A, 38 patients were classified Child–Pugh class B, and 13 patients were classified Child–Pugh class C. There were significantly different variables in etiology of cirrhosis, laboratory findings (including sodium, platelet, albumin, total bilirubin, AST, INR), fECV, FIB4, APRI and MELD between Child–Pugh Classes (Table 1).

### 3.2. Correlation and Discrimination of Noninvasive Markers by Liver Function

**Figure 3:**
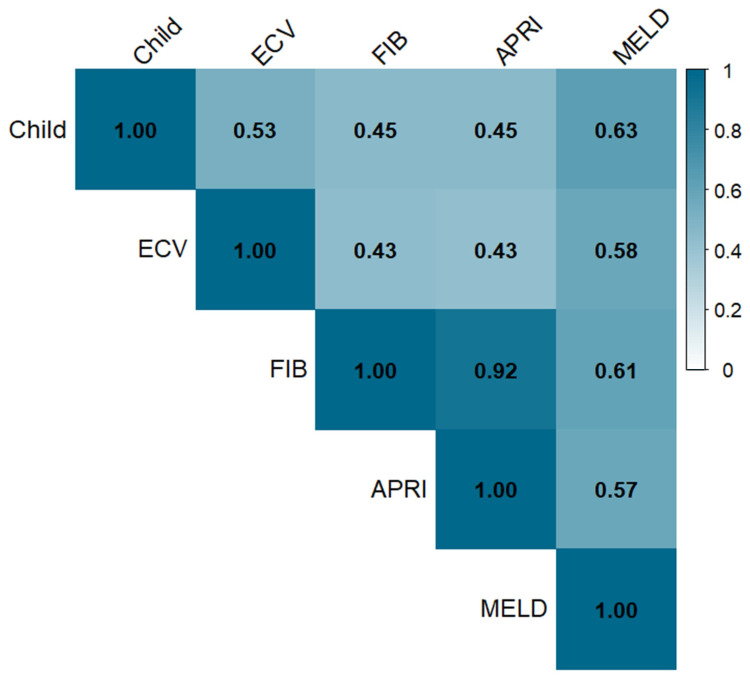
Heatmap of Spearman correlation coefficients between Child–Pugh class and noninvasive markers. (Abbreviations: CP class, Child–Pugh classification; fECV, extracellular volume fraction; FIB-4, fibrosis-4 index; APRI, aspartate aminotransferase to platelet ratio index; MELD, model for end-stage liver disease).

The mean values of four noninvasive markers showed significant differences among the three Child–Pugh classes (*p* < 0.001, Table 2). In addition, all four noninvasive markers were positively correlated with the Child–Pugh classification in Spearman’s rank test (Figure 3).

To further explore the differences in noninvasive marker values among Child–Pugh classes, a Games–Howell post hoc analysis was performed. fECV and MELD showed significant mean differences between both Child–Pugh A vs. B and B vs. C (Table 3). However, FIB-4 and APRI demonstrated significant mean differences only between Child–Pugh A vs. B (Table 3).

The intra- and interobserver reliabilities of the fECV measurements were excellent (ICC, 0.93; 95% confidence interval (CI), 0.91–0.95 and 0.91; 95% CI, 0.88–0.92, respectively).

### 3.3. Predictive Value of Noninvasive Markers for Liver Function

**Figure 4:**
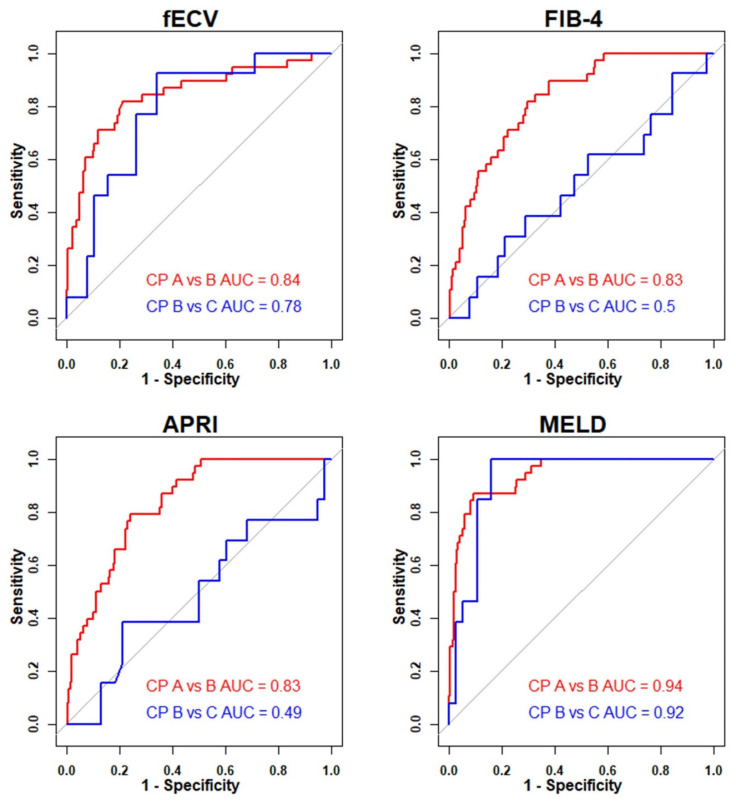
Diagnostic performance of noninvasive markers in classifying Child–Pugh classes. (Abbreviations: CP class, Child–Pugh classification; AUC, area under the receiver operating characteristic curve; fECV, extracellular volume fraction; FIB-4, fibrosis-4 index; APRI, aspartate aminotransferase to platelet ratio index; MELD, model for end-stage liver disease).

The AUC of fECV for differentiating Child–Pugh A from B was 0.84 (95% CI, 0.76–0.92). There was no significant difference compared to FIB-4 (0.83; 95% CI, 0.77–0.89; *p* > 0.99) or APRI (0.83; 95% CI, 0.77–0.89; *p* > 0.99). MELD showed a significantly higher AUC (0.94; 95% CI, 0.90–0.97; *p* = 0.047) than fECV for differentiating Child–Pugh A from B. For differentiating Child–Pugh B from C, fECV (0.78; 95% CI, 0.65–0.92) demonstrated superior AUCs compared to FIB-4 (0.51; 95% CI, 0.31–0.70; *p* = 0.038) and APRI (0.51; 95% CI, 0.32–0.71; *p* = 0.037). Although MELD showed a higher AUC (0.92; 95% CI, 0.85–1.00; *p* = 0.116) than fECV for differentiating Child–Pugh B from C, the difference was not statistically significant. Figure 4 illustrates the receiver operating characteristic curves of noninvasive markers for differentiating Child–Pugh classes, and Table 4 summarizes the comparison of AUCs between fECV and other markers.

## 4. Discussion

In this study, we evaluated the correlation between fECV and liver function, and compared it with other noninvasive biomarkers. The fECV, derived from the delayed phase (180 s) of dual-source DECT and corrected using hematocrit and aortic enhancement, showed a positive correlation with worsening liver function as indicated by higher Child–Pugh classifications. Notably, fECV values significantly increased between Child–Pugh classes B and C, a pattern not observed for FIB-4 or APRI. These results suggest that the corrected extracellular volume fraction of the liver is a more reliable indicator of hepatic functional reserve than composite markers based on age, AST, ALT, and platelet count. And fECV may be a more sensitive marker for detecting incremental deterioration in hepatic functional reserve. This finding is consistent with previous studies demonstrating the utility of fECV in assessing liver function [29,32].

The fECV values in this study were derived from iodine maps obtained using DECT, which allows for the direct quantification of iodine concentration in both the liver parenchyma and blood pool. This approach likely enhanced the correlation between fECV and liver function compared to conventional single-energy CT techniques. Previous studies have reported that fECV derived from DECT more accurately predicts liver fibrosis staging and prognosis in patients with cirrhosis and impaired liver function than values obtained using single-energy CT methods [21,33,34]. This superiority is attributable to the ability of DECT to directly measure iodine content, thereby minimizing the influence of confounding tissue components. Unlike single-energy CT, which relies on attenuation differences between pre- and post-contrast images, DECT provides a more accurate and robust estimation of fECV. Furthermore, DECT eliminates the risk of image misregistration, may occur when pre- and post-contrast images are acquired in separate phases, thereby improving the precision of fECV measurements. Importantly, as precontrast imaging is not required, DECT also offers the advantage of reducing radiation exposure in clinical settings. Collectively, these technical advantages improve the reliability of DECT-derived fECV as a noninvasive biomarker for assessing liver function. Furthermore, as reported by Megan et al., the 100/140 Sn kVp setting demonstrated significantly greater accuracy for iodine quantification compared to the 80/140 Sn kVp setting in a large abdominal phantom [35]. Based on these results, the 100/140 Sn kVp setting used in our study may not only enhance the precision and reliability of iodine measurements but also suggest its applicability to kVp settings used in daily clinical practice. Additionally, we utilized 3 min delayed images, which are routinely included in multiphase liver CT examinations in clinical practice. Therefore, we believe that the DECT-based fECV score derived using this approach can serve as a robust and reliable marker for the follow-up of patients with liver cirrhosis in daily clinical settings.

While MELD showed significantly better diagnostic performance than fECV in differentiating between Child–Pugh class A and B in our study, this finding contrasts with previous studies that directly compared MELD and fECV [29]. This discrepancy may be attributed to several factors, including heterogeneity in study populations, differences in the methods used for fECV correction, and the use of various versions of the MELD scoring system. Importantly, prior research has suggested that fECV may be more reflective of advanced hepatic fibrosis or liver function deterioration, rather than subtle or early-stage changes [7,29,34,36]. The fact that the significant difference between MELD and fECV was observed only in the early-stage comparison (Child–Pugh A vs. B) may reflect this inherent characteristic of fECV. Given the absence of a universally accepted gold standard for evaluating hepatic functional reserve, we used the Child–Pugh score as a reference in this study. Notably, the serum markers used in the Child–Pugh score calculation are also incorporated in the MELD formula. This overlap may partly explain why MELD exhibited stronger correlations and higher AUC values than fECV in relation to Child–Pugh classification. From a mechanistic standpoint, fECV reflects anatomical changes in the liver parenchyma, whereas MELD is derived from laboratory parameters that represent functional impairment, including renal function, which may render MELD more sensitive to changes in hepatic functional reserve [15,17,18,19,20,24,37]. Given the limited number of studies directly comparing MELD and fECV, further investigation is warranted to clarify their relative performance in different clinical contexts. Moreover, the MELD score, while primarily used for prioritizing liver transplantation, has limitations in that it can be influenced by therapeutic interventions such as diuretics, as well as by clinical conditions like sepsis or hemolysis [23,24]. Therefore, the existence of an additional tool that can effectively estimate liver functional reserve in patients with liver cirrhosis is meaningful, not only in enhancing prognostic accuracy but also in complementing the inherent limitations of MELD.

The ability to calculate fECV using previously acquired CT scans, without the need for additional procedures, highlights its clinical potential. Since the measurement of ROI in the liver and aorta on CT is simple and time-efficient, integrating fECV values into the radiologic interpretation of routine diagnostic or screening CT scans could assist in estimating a patient’s hepatic functional reserve. Furthermore, serial assessment of fECV over time may help detect subtle changes associated with disease progression. Tracking these serial changes via CT may be particularly valuable in patients with obesity or ascites, where ultrasound or MRI is limited. With recent advances in artificial intelligence, rapid, accurate, and fully automated quantitative analysis is becoming increasingly feasible. Leveraging these technologies may further enhance the practicality and precision of fECV assessment as an additional tool for clinical decision-making.

Our study has several limitations. First, because this was a retrospective study conducted at a single institution, there may be inevitable selection bias. Second, while iodine quantification results were consistent in our study, they may vary depending on the type of DECT system used—such as dual-source, rapid kVp switching, dual-layer detector, or split-filter systems—as well as the specific scanner and kVp settings. Third, the relatively small number of patients with Child–Pugh class C compared to classes A and B may limit the statistical power of our findings. However, it is worth noting that our study included a larger number of patients than previous studies addressing similar topics [21,30]. Fourth, only a small number of patients were pathologically confirmed to have cirrhosis. Therefore, patients with early cirrhosis who may show negative clinical findings and CT imaging features indicative of cirrhosis might have been excluded.

## 5. Conclusions

Our study revealed a significant association between fECV and liver function reserve, as assessed by Child–Pugh class. Furthermore, fECV could distinguish between a sustained and a decreased liver functional reserve (Child–Pugh A vs. B), as well as between moderately and severely decreased reserves (Child–Pugh B vs. C).

The findings of our study revealed that the extracellular volume fraction (fECV) of the liver derived from dual-source dual-energy CT may serve as a noninvasive marker for predicting hepatic functional reserve. Furthermore, it has the potential to be used as a longitudinal parameter for monitoring disease progression over time. Given its distinct measurement characteristics, fECV may also play a complementary role alongside other noninvasive biomarkers in the comprehensive assessment of liver function.

## Figures and Tables

**Table 1:** Baseline characteristics by Child–Pugh class.

Variable	Child–Pugh A (*n* = 207)	Child–Pugh B (*n* = 38)	Child–Pugh C (*n* = 13)	*p* Value
Age	58.0 [52.0; 65.0]	55.0 [48.0; 70.0]	55.0 [50.0; 57.0]	0.174
Sex				0.67
Male	150 (72.5%)	30 (78.9%)	9 (69.2%)	
Female	57 (27.5%)	8 (21.1%)	4 (30.8%)	
Sodium	141.3 [140.1; 142.8]	139.1 [135.1; 140.4]	134.1 [132.5; 139.0]	**< 0.001**
Etiology				**0.001**
Alcohol	84 (40.6%)	29 (76.3%)	10 (76.9%)	
HBV	76 (36.7%)	2 (5.3%)	1 (7.7%)	
HCV	35 (16.9%)	4 (10.5%)	2 (15.4%)	
Autoimmune	2 (1.0%)	1 (2.6%)	0 (0.0%)	
Unknown	10 (4.8%)	2 (5.3%)	0 (0.0%)	
Platelet	122.0 [87.0; 165.5]	72.0 [50.0; 95.0]	64.0 [51.0; 112.0]	**< 0.001**
Albumin	4.4 [4.1; 4.7]	3.2 [3.0; 3.7]	2.6 [2.4; 3.1]	**< 0.001**
Bilirubin	0.9 [0.7; 1.3]	2.7 [1.3; 3.6]	4.8 [3.6; 5.5]	**< 0.001**
AST	29.0 [24.0; 41.5]	55.0 [38.0; 101.0]	48.0 [41.0; 78.0]	**< 0.001**
ALT	22.0 [17.0; 31.0]	25.0 [20.0; 39.0]	29.0 [17.0; 34.0]	0.209
Creatinine	0.8 [0.7; 0.9]	0.7 [0.6; 1.0]	0.7 [0.6; 1.0]	0.884
INR	1.1 [1.1; 1.2]	1.4 [1.2; 1.5]	1.9 [1.8; 2.0]	**< 0.001**
fECV	23.6 [20.6; 26.3]	32.8 [27.2; 38.7]	39.4 [37.2; 43.3]	**< 0.001**
FIB-4	3.2 [2.0; 5.5]	9.5 [5.2; 13.1]	9.3 [5.1; 13.3]	**< 0.001**
APRI	0.7 [0.4; 1.3]	2.2 [1.4; 5.5]	2.2 [1.1; 3.6]	**< 0.001**
MELD	8.0 [6.9; 9.6]	14.0 [12.6; 16.7]	22.1 [21.1; 24.4]	**< 0.001**

Values are expressed as No. (%) or median [IQR] as appropriate. Bold values indicate statistical significance (*p* < 0.05). (Abbreviations: HBV, hepatitis B virus; HCV, hepatitis C virus; AST, aspartate aminotransferase; ALT, alanine aminotransferase; INR, international normalized ratio; fECV, extracellular volume fraction; FIB-4, fibrosis-4 index; APRI, aspartate aminotransferase to platelet ratio index; MELD, model for end-stage liver disease).

**Table 2:** Comparison of noninvasive markers by Child–Pugh class.

Child–Pugh Class	Mean Value of Noninvasive Markers (Mean ± SD)				N
	fECV	FIB-4	APRI	MELD	
A	23.93 ± 5.05	4.68 ± 5.86	1.23 ± 1.58	8.49 ± 2.13	207
B	32.81 ± 7.41	11.38 ± 9.35	3.28 ± 2.74	14.74 ± 4.14	38
C	40.23 ± 6.63	11.88 ± 11.21	3.75 ± 3.73	22.79 ± 3.19	13
*p* value *	<0.001	<0.001	<0.001	<0.001	

(Abbreviations: SD, standard deviation; fECV, extracellular volume fraction; FIB-4, fibrosis-4 index; APRI, aspartate aminotransferase to platelet ratio index; MELD, model for end-stage liver disease). * *p* values were calculated using Welch’s one-way analysis of variance.

**Table 3:** Pairwise differences in noninvasive markers between Child–Pugh classes.

Child–Pugh Class	Mean Difference in Noninvasive Markers [95% CI]			
	fECV	FIB-4	APRI	MELD
A vs. B	8.88 *** [5.85, 11.92]	6.70 *** [2.89, 10.52]	2.05 *** [0.94, 3.16]	6.25 *** [4.58, 7.92]
B vs. C	7.42 ** [1.92, 12.91]	0.50 [–8.33, 9.32]	0.47 [–2.42, 3.36]	8.05 *** [5.29, 10.80]

(Abbreviations: CI, confidence interval; fECV, extracellular volume fraction; FIB-4, fibrosis-4 index; APRI, aspartate aminotransferase to platelet ratio index; MELD, model for end-stage liver disease). ** *p* = 0.007, *** *p* < 0.001 based on Games–Howell post hoc tests after Welch’s one-way analysis of variance.

**Table 4:** Comparison of AUC differences between fECV and other noninvasive markers.

Child–Pugh Class	fECV vs. APRI		fECV vs. FIB-4		fECV vs. MELD	
	*p* Value	95% CI	*p* Value	95% CI	*p* Value	95% CI
A vs. B	>0.99	−0.08, 0.10	>0.99	−0.08, 0.10	**0.047**	−0.18, −0.02
B vs. C	**0.037**	0.07, 0.52	**0.038**	−0.06, 0.50	0.116	−0.28, 0.01

Bold values indicate statistical significance (*p* < 0.05). *p* values were calculated using DeLong’s test with Holm correction for multiple comparisons (Abbreviations: AUC, area under the receiver operating characteristic curve; fECV, extracellular volume fraction; FIB-4, fibrosis-4 index; APRI, aspartate aminotransferase to platelet ratio index; MELD, model for end-stage liver disease; CI, confidence interval).

## Data Availability

The datasets generated during and/or analyzed during the current study are available from the corresponding author on reasonable request.
